# Supplementary material for: Alzheimer’s Disease Caregiver Characteristics and Their Relationship with Anticipatory Grief
Source: Int J Environ Res Public Health. 2021 Aug 22;18(16):8838. doi: 10.3390/ijerph18168838 (PMC8392352; doi:10.3390/ijerph18168838)
Supplement: Supplementary file 1 [file ijerph-18-08838-s001.zip › ijerph-1312829-supplementary.pdf]

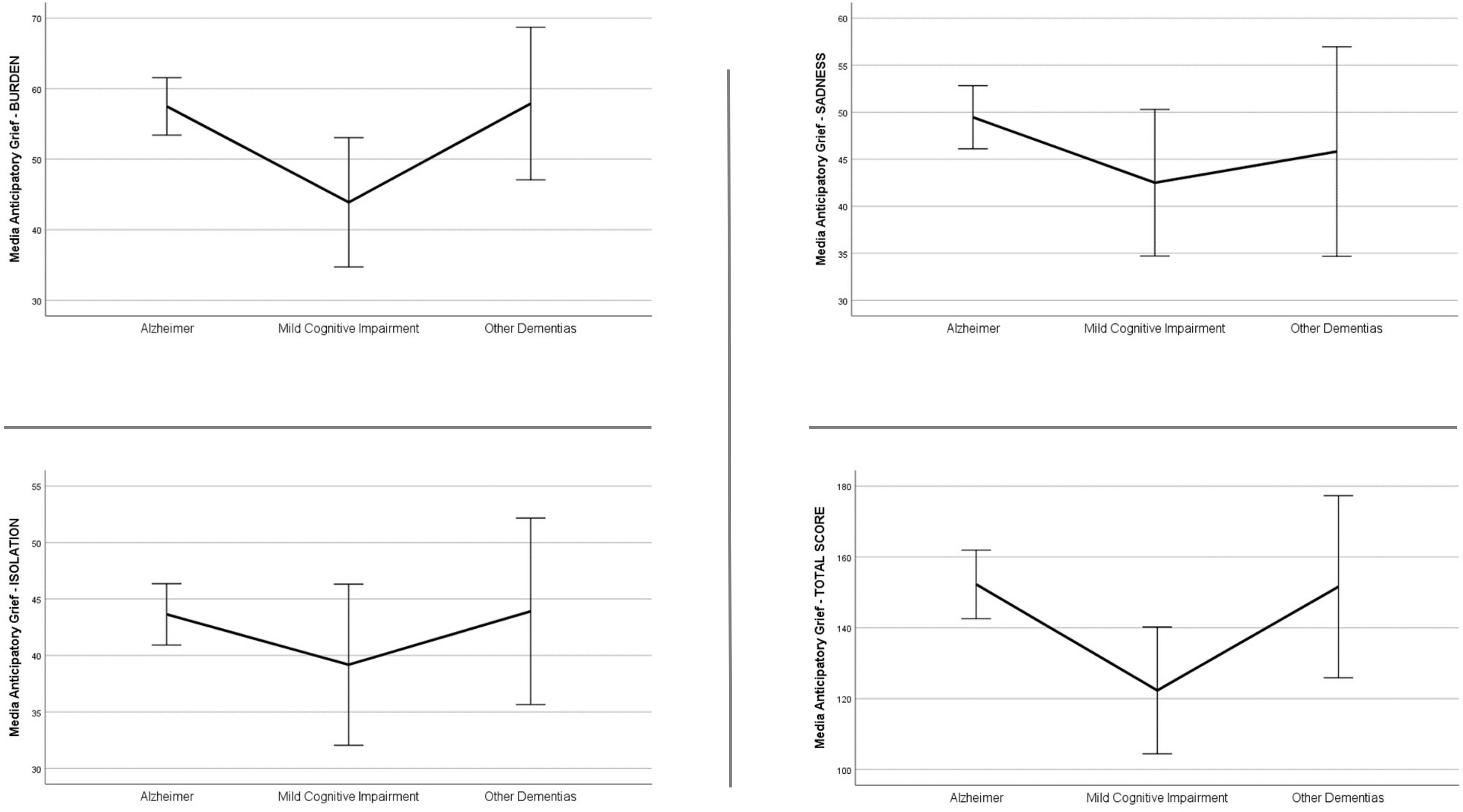

Figure S1. Effect of the diagnosis on the dimensions of the anticipatory grief component of the MM Caregiver Inventory.
